# Regulator of G Protein Signaling 3 Modulates Wnt5b Calcium Dynamics and Somite Patterning

**DOI:** 10.1371/journal.pgen.1001020

**Published:** 2010-07-08

**Authors:** Christina M. Freisinger, Rory A. Fisher, Diane C. Slusarski

**Affiliations:** 1 Department of Biology, University of Iowa, Iowa City, Iowa, United States of America; 2 Department of Pharmacology, University of Iowa College of Medicine, Iowa City, Iowa, United States of America; University of North Carolina, United States of America

## Abstract

Vertebrate development requires communication among cells of the embryo in order to define the body axis, and the Wnt-signaling network plays a key role in axis formation as well as in a vast array of other cellular processes. One arm of the Wnt-signaling network, the non-canonical Wnt pathway, mediates intracellular calcium release via activation of heterotrimeric G proteins. Regulator of G protein Signaling (RGS) proteins can accelerate inactivation of G proteins by acting as G protein GTPase-activating proteins (GAPs), however, the possible role of RGS proteins in non-canonical Wnt signaling and development is not known. Here, we identify *rgs3* as having an overlapping expression pattern with *wnt5b* in zebrafish and reveal that individual knockdown of either *rgs3* or *wnt5b* gene function produces similar somite patterning defects. Additionally, we describe endogenous calcium release dynamics in developing zebrafish somites and determine that both *rgs3* and *wnt5b* function are required for appropriate frequency and amplitude of calcium release activity. Using rescue of gene knockdown and *in vivo* calcium imaging assays, we demonstrate that the activity of Rgs3 requires its ability to interact with Gα subunits and function as a G protein GAP. Thus, Rgs3 function is necessary for appropriate frequency and amplitude of calcium release during somitogenesis and is downstream of Wnt5 activity. These results provide the first evidence for an essential developmental role of RGS proteins in modulating the duration of non-canonical Wnt signaling.

## Author Summary

Vertebrate development requires communication among cells in order to define the body axis (front/back, head/tail, or left/right). Secreted factors such as Wnts play key roles in a vast array of cellular processes, including patterning of the body axis. One arm of the Wnt-signaling network, the non-canonical pathway, mediates intracellular calcium release via activation of heterotrimeric G proteins. Regulator of G protein Signaling (RGS) proteins can accelerate inactivation of G proteins by acting as G protein GAPs and are uniquely situated to control the amplitude of a Wnt signal. Here, we combine cellular, molecular, and genetic analyses with high resolution calcium imaging to identify a role for RGS modulation of Wnt-mediated calcium release dynamics and developmental patterning events. We find that loss of *rgs3* gene function produced body patterning defects like those observed with loss of wnt5b gene function. Analysis of endogenous calcium release dynamics in developing zebrafish revealed that both rgs3 and wnt5b are required for appropriate frequency and amplitude of calcium release. Our results provide new evidence that a member of the RGS protein family is essential for modulating the non-canonical Wnt network to assure normal tissue patterning during development.

## Introduction

The Wnt signaling network is classified into β-catenin-dependent and β-catenin-independent pathways [1]–[3]. β-catenin-dependent Wnt signaling acts through the stabilization of β-catenin and subsequent transcriptional activation of β-catenin targets [4], whereas β-catenin-independent Wnt signaling influences cell polarity (known as Planar Cell Polarity or PCP in *Drosophila*). β-catenin-independent Wnt signaling has also been shown to lead to calcium (Ca^2+^) release as well as activation of Rac, Rho and other cytoskeletal components in vertebrates [5], [6]. In zebrafish, Wnt-5 and -11 function in Wnt/Ca^2+^ signaling [7], [8]. Wnt11 is enriched in the anterior and mutants show anterior extension and eye fusion defects, while Wnt5b is enriched in the posterior and mutants show altered cell movements during gastrulation, often resulting in convergence extension and somite defects [9]–[11].

Zebrafish embryos demonstrate Ca^2+^ release dynamics during epiboly, gastrulation, convergent extension and organogenesis [12]–[21]. Two distinct types of Ca^2+^ release events, aperiodic transient fluxes found mainly in the enveloping layer and dorsal forerunner cells [17], [18], [22], [23] and sustained increases in Ca^2+^ levels in the deep cell layer and yolk syncytial layer [24], [25], have been described. We have shown that early Ca^2+^ transients are, in part, modulated by Wnt5 [15], [26]. The zebrafish *wnt5b* genetic mutant (*pipetail*) shows reduced Ca^2+^ release [24] and the ventralized maternal effect mutant *hecate* shows ectopic Ca^2+^ release [18]. Moreover, inhibition of Ca^2+^ release results in alteration of dorsal ventral patterning, cell movement and left-right patterning [17], [26]. These observations suggest that the kinetics of Ca^2+^ release, both transient and sustained, translate into distinct developmental outputs [16].

Wnts interact with receptors of the Frizzled (Fz) family [27] and due to structural similarities to G protein coupled receptors (GPCR), Fz receptors are hypothesized to stimulate heterotrimeric G protein activation [28]–[30]. We have shown previously that Wnt proteins work though specific Fz homologues to activate G proteins and to modulate Ca^2+^ release in zebrafish embryos [15], [22], [26], [31]. Moreover, in Drosophila, Wnt target genes have been shown to be upregulated when Gαo is over-expressed and constitutively active Gαo is sufficient to restore Wnt signaling in the absence of Fz activity [32]. In addition, epistasis experiments support that G proteins function downstream of Fz and upstream of Disheveled (Dvl) [32].

G protein signaling is regulated by the lifetime of active Gα and βγ subunits. Activated Gα subunits have an intrinsic GTPase activity that converts the GTP-bound conformation to the Gα-GDP bound conformation allowing reassembly with Gβγ subunits to form the inactive Gαβγ heterotrimer [33]. Regulator of G protein Signaling (RGS) proteins have been shown to influence the duration of G protein signaling [34]–[37]. RGS proteins share a conserved RGS domain of 130 amino acids that binds to activated Gα subunits and accelerates their rates of GTP hydrolysis by up to 1000-fold [38]–[40]. By functioning as GTPase-activating proteins (GAPs) for G proteins, RGS proteins are uniquely situated to modulate the intensity and duration of Wnt signaling. However, no studies have ascertained the possible importance of RGS proteins in non-canonical Wnt signaling or whether changes in frequency and or amplitude of signaling result in biological defects.

To investigate potential roles of RGS proteins in vertebrate development, we utilize gene knockdown in zebrafish. We focus on *rgs*3, which was identified in an expression screen in zebrafish [41]. We find that *rgs3* is expressed in overlapping and adjacent domains with *wnt5b* at multiple stages of zebrafish development. Morpholino knockdown of *rgs3* in zebrafish embryos causes convergence and extension (CE) defects that resemble phenotypes observed in the *wnt5b* genetic mutant, *pipetail* [42]. To this end, we have identified a genetic interaction between *rgs3* and *wnt5b*. Additionally, we describe endogenous Ca^2+^ release dynamics during somite stages and show that Rgs3 and Wnt5b impact the frequency of Ca^2+^ release. Moreover, we show that Rgs3 modulates the extent and duration of Wnt5b induced Ca^2+^ activity. Functional analyses show that both the rescue of the *rgs3* knockdown defect and impact on Wnt5-induced Ca^2+^ release requires a key asparagine in the RGS domain of Rgs3 necessary for Gα binding and acceleration of its GTPase activity. This research identifies a link between Wnt5b signaling and Rgs3 activity relative to the frequency of Ca^2+^ release, thus revealing obligatory roles for RGS proteins in vertebrate development in the context of the whole animal. Our results also demonstrate that the biological outcome of Wnt signaling depends greatly upon regulating the duration of its signal, as shown here with Rgs3.

## Results

### Expression of *rgs3*

**Figure 1:**
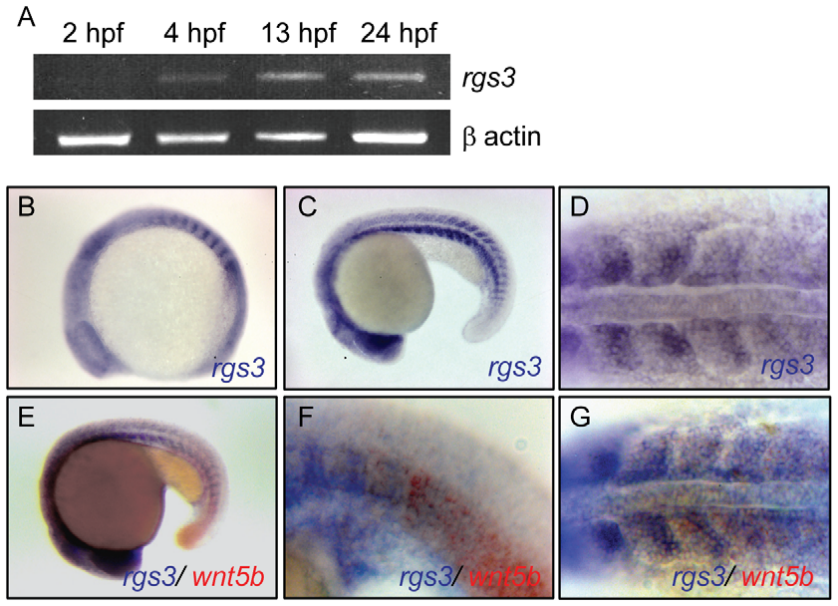
Temporal and spatial expression of *rgs3* throughout zebrafish development. RT-PCR was used to determine the temporal expression of *rgs3* from 0 hpf to 24 hpf (A). Whole Mount *In Situ* Hybridization was utilized to determine the spatial expression of *rgs3* in 12 hpf (B) and 20hpf (C,D) wild type embryos. *rgs3* and *wnt5b* double label *in situ* in18hpf embryos (E–G). Lateral (B,C,E,F) and dorsal (D,G) views illustrate that *rgs3* is expressed in the developing somites (B–D) and posterior tail (C). At 18 hpf *rgs3* expression is enriched in the posterior (caudal) portion of the developing somites (D). Co-localization of *wnt5b* and *rgs3* was determined by double label WMISH with *wnt5b* (red) and *rgs3* (blue) showing overlapping expression domains in the developing tail and somites (E–G). Sense probes (negative control) gave no specific hybridization signal.

Zebrafish *rgs3* was identified in an expression screen during early somitogenesis stages [41] and is poised to interact with the Wnt signaling network. Utilizing Reverse Transcriptase Polymerase Chain Reaction (RT-PCR), we determined that *rgs3* expression begins during the blastula period shortly after zygotic transcription initiates (2.5–5 hours post fertilization, hpf), and persists through the segmentation period (10–24hpf) (Figure 1A). Whole Mount *In Situ* Hybridization (WMISH) demonstrated ubiquitous *rgs3* expression during epiboly and gastrulation stages. During somite stages (10–20 hpf), *rgs3* expression resolves in the somites, tailbud, and brain (Figure 1B–1G), with discrete *rgs3* expression in the midbrain/hindbrain boundary as demonstrated by overlap with the molecular marker engrailed 1 (*eng1*) at 28 hpf (Figure S1F), and enriched *rgs3* expression in the posterior (caudal) portion of developing somites (Figure 1D). *rgs3* and *wnt5b* show both overlapping and adjacent expression domains in the somites and in the posterior tailbud (Figure 1E–1G and Figure S1A, S1B, S1C, S1D). *rgs3* expression is enriched around the Kupffer's vesicle (Figure S1C), a ciliated organ in the zebrafish embryo that has been shown to influence left-right patterning, yet *rgs3* does not appear to be required for organ laterality (data not shown). As Wnt5b is a secreted ligand, the proximity of *rgs3* to *wnt5b* producing cells suggests that Rgs3 may function in modulating Wnt5b signaling.

### Rgs3 is sufficient to suppress Wnt5b-induced Ca^2+^ dynamics

**Figure 2:**
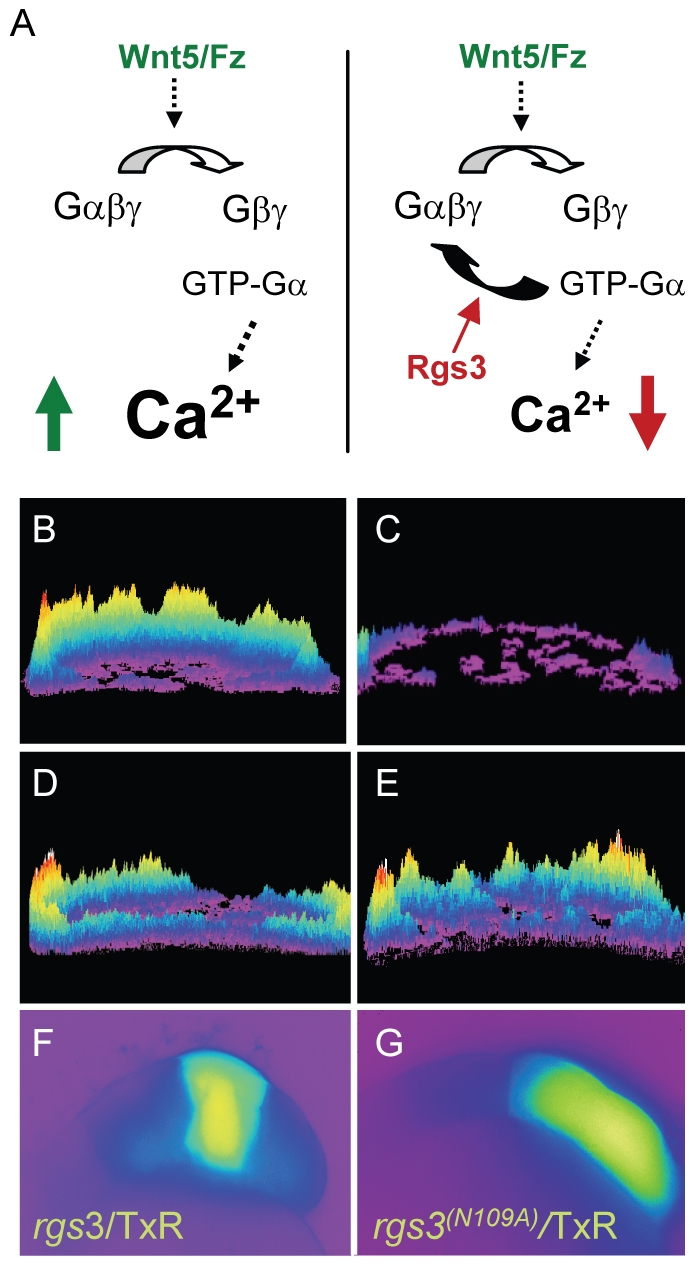
*rgs3* inhibits *wnt5b-*induced Ca^2+^ dynamics. Schematic representation illustrating that Wnt5b overexpression results in intracellular calcium release in a G protein dependent manner (A, left side) and the predicted negative effect overexpression of Rgs3 will have on the Wnt/calcium pathway (A, right side). Representative Ca^2+^ release profiles (composite image) of *wnt5-*overexpressing (B,D,E) and wt (C) blastula stage zebrafish embryos. (B–E) are composites of fura-2 ratiometric imaging time course showing total calcium release activity as peaks and colors mapped topographically. Ca^2+^ release profile of an embryo uniformly expressing *wnt5b* (B). Wt Ca^2+^ release profile (C). *wnt5b* expressing embryo with localized TxR/*rgs3* (D) or Txr/*rgs3* ^N109A^ (E). Corresponding fluorescent images illustrate the location of TxR/*rgs3* (F) and TxR/*rgs3^N109A^* (G).

In zebrafish, *wnt5b* induces increased Ca^2+^ release during the blastula stage in a G protein dependent manner [15], [22], [26]. To determine if *rgs3* over-expression is sufficient to negatively regulate Wnt5b signaling (Figure 2A), we tested the impact of *rgs3* on *wnt5b* induced Ca^2+^ release. In vivo imaging in blastula stage embryos is accomplished with the Ca^2+^ sensor Fura-2. Upon binding Ca^2+^, Fura-2 exhibits an absorption shift that can be determined by collection at two wavelengths (340nm, Ca^2+^-saturated and 380nm, Ca^2+^-free). A ratio image is derived as the quotient of the 340-nm image divided by the 380-nm image on a pixel-by-pixel basis, and provides spatial and temporal measurement of Ca^2+^ dynamics. Ca^2+^ release activity was monitored over a 75 minute time course during the blastula stage. Sequential ratiometric images were analyzed by a subtractive algorithm to identify changes in Ca^2+^ release activity (transients) over time as well as the location of the activity as described previously [13], [43]–[45]. Transients identified during the time course are presented as a composite image with location of Ca^2+^ release mapped on the embryo. The number of Ca^2+^ transients during the cellular blastoderm stage is represented by height of the peaks and color coded where purple is low and yellow/red is high number of events. The composite image of a wild-type (wt) embryo during the blastula stage indicates endogenous Ca^2+^ levels throughout the embryo (Figure 2C) compared to those observed during increased Ca^2+^ release in an embryo injected with *wnt5b* (Figure 2B). Co-injection of *rgs3* with dextran-conjugated Texas Red (TxR) lineage tracer into a subset of cells in embryos uniformly expressing *wnt5b* co-mixed with Fura-2 supports that *rgs3* is sufficient to suppress *wnt5b* induced Ca^2+^ release as demonstrated by the reduction of Ca^2+^ levels (Figure 2D) in the *rgs3*/TxR positive region (Figure 2F).

**Figure 3:**
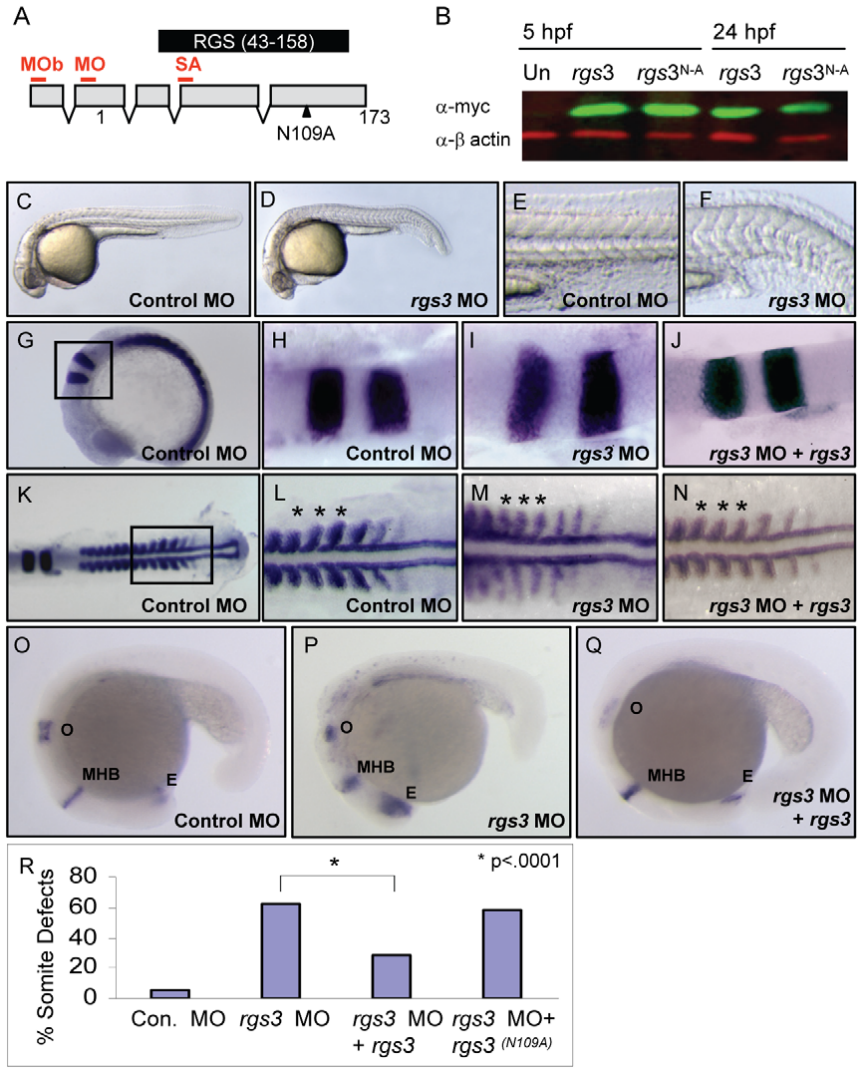
*rgs3* morphant phenotypes and functional rescue. Schematic of zebrafish rgs3 mRNA/protein composite (A). Numbers refer to the amino acid number of the encoded Rgs3 protein, while the locations of morpholino binding sites employed in later experiments are indicated by red lines above the transcript. MO = rgs3 MO, MOb = rgs3 MOb and SA = rgs3 MOsa. The RGS domain of Rgs3, amino acids 43–158, is highlighted by the black box (A). Western analysis demonstrates that myc-tagged rgs3 and rgs3^N109A^ proteins are detectable from 5 hpf to 24 hpf (B). Antisense morpholino-mediated gene knockdown of *rgs*3 results in embryonic defects. Lateral views of 28 hpf wild-type (C,E) and *rgs3* MO injected (D,F) embryos illustrate that *rgs3* morphants have a reduced body length (D) and altered somite formation (F). *rgs3* was co-injected with *rgs3* MO to monitor rescue of gene knockdown. The molecular markers *krox20*, *myoD* and *pax2* were used to evaluate rgs3 morphant rescue (G–Q). *kro*x 20 labels rhombomeres 3 and 5, *myoD* labels the developing somites and adaxial cells while, *pax2* labels the otic vesicle (o), midbrain-hindbrain boundary (MHB) and eye (E). Lateral (G and O–Q) and dorsal (H–N) views, anterior to the right, of 15 hpf (G–N) and 20 hpf (O–Q) wt embryos injected with Control MO (G,H,K,L,O), *rgs3* MO (I,M,P) and *rgs3* MO+*rgs*3 (J,N,Q). Boxed regions in G and K represent the areas magnified in H–J and L–N respectively. Asterisks indicate the spacing and width of three representative somites (L–N). *krox20*, *myoD* and *pax2* expression patterns indicate that *rgs3* is able to suppress the morpholino-induced defect (J,N,Q). For structural functional analyses, *rgs*3^N109A^ was evaluated for rescue of knockdown. Morphological analyses reveals that *rgs3* is able to suppress the MO induced defect (R) while, *rgs3* ^(N109A)^ is unable to suppress the MO induced defect (R).

We next investigated if Rgs3 suppression of *wnt5b* induced Ca^2+^ release requires GAP activity. A conserved asparagine within the RGS domain of RGS proteins is necessary for GAP activity for Gα subunits [46]–[48]. Substitution of this key asparagine (N) with Alanine (A) results in a loss of the GAP activity of RGS proteins towards Gα subunits in culture cells [46], [48]. To elucidate the role of the GAP function of Rgs3, we created an N to A mutation in zebrafish *rgs3* (*rgs*3^N109A^)(Figure 3A). We evaluated the impact of *rgs*3^N109A^ expression on Wnt5b induced Ca^2+^ release. Unlike *rgs3*, the *rgs*3^N109A^ is unable to suppress *wnt5b* induced Ca^2+^ release (Figure 2E) as demonstrated by no change in the Ca^2+^ activity in the *rgs*3^N109A^ /TxR positive region of embryos (Figure 2G). To rule out the possibility that lack of suppression by Rgs3^N109A^ was due to differences in its expression or localization compared to Rgs3, we generated and expressed N-terminal myc-tagged *rgs*3 and *rgs*3^N109A^ constructs in embryos. Western analysis reveals robust and comparable expression of Rgs3 and Rgs3^N109A^ at the time of Ca^2+^ imaging as well as through 24hpf (Figure 3B). Immunostaining for anti-myc in epiboly stage embryos also indicates that both proteins localize to the membrane and cytoplasm (Data not shown). Together these data strongly indicate that *rgs3* is sufficient to inhibit *wnt5*b-induced Ca^2+^ signaling and that this action requires the GAP activity of Rgs3.

### Endogenous requirement for *rgs3* during embryogenesis

Since Rgs3 is sufficient to modulate Wnt5 activity in an over-expression assay, we next evaluated the necessary role of *rgs3* during development. To knockdown Rgs3, we utilized antisense morpholino oligonucleotides (MO) [49]. Three separate MOs were designed to bind *rgs3* 5′UTR (MO and MOb) or splice junction (SA) (Figure 3A). All MOs designed to knockdown Rgs3 produced similar defects. Control-injected embryos at 28 hpf are fully extended with a characteristic anterior-posterior (A-P) length (Figure 3C). In contrast, *rgs3* MO-injected embryos have shorter A-P extension and a kinked tail (Figure 3D) reminiscent of defects observed in the *wnt5b* (*pipetail*) genetic mutant [42]. Zebrafish somites develop sequentially anterior to posterior and form a distinct chevron shape [50] (Figure 3E). *rgs3* morphants display tighter packed and rounded somites (Figure 3F). To evaluate anterior-posterior extension alterations at an earlier developmental stage (15 hpf), molecular markers were used. Control-injected embryos have a characteristic spacing of *krox20* expression in the hindbrain rhombomeres 3 and 5, as well as regular spaced blocks of *myoD* expression in the developing somites flanking the midline (Figure 3G–3H and 3K–3L). In contrast, *krox20* and *myoD* expression in *rgs3* morphants reveal a failure of cells to converge on the midline resulting in a lateral expansion of the rhombomeres and somites (Figure 3I and 3M). Additionally, *rgs3* morphants fail to extend along the anterior-posterior (A-P) axis leading to closer spaced *myoD* (Figure 3M, asterisks). The A-P extension defects were further confirmed with *pax2*, a marker expressed in the anterior retina, midbrain/hindbrain, and otic vesicle of 18 hpf embryos (Figure 3O). *rgs3* morphants display compression of these regions along the A-P axis (Figure 3P). Together these data strongly indicate that *rgs3* is required for normal anterior-posterior axis extension.

The specificity of the rgs3 knockdown as well as structural functional analyses were determined by RNA co-injection experiments. Injection of control 5bp mismatch MO resulted in negligible defects compared to *rgs3* MO which induced morphological somite defects (Figure 3R). Co-injection of *rgs3* MO with *rgs*3 RNA suppressed the MO-induced defects evaluated by molecular markers *krox20* (Figure 3J), *myoD* (Figure 3N, asterisks) and *pax2* (Figure 3Q). Moreover, wild-type *rgs3* RNA leads to significant suppression of MO-induced defects (Figure 3R and Table S1). In contrast, *rgs*3^N109A^ mutant RNA does not suppress the MO-induced defect (Figure 3R and Table S1). These results demonstrate that Rgs3 GAP activity is required for its developmental functions.

### *rgs3* function is necessary for endogenous Ca^2+^ dynamics in somites

**Figure 4:**
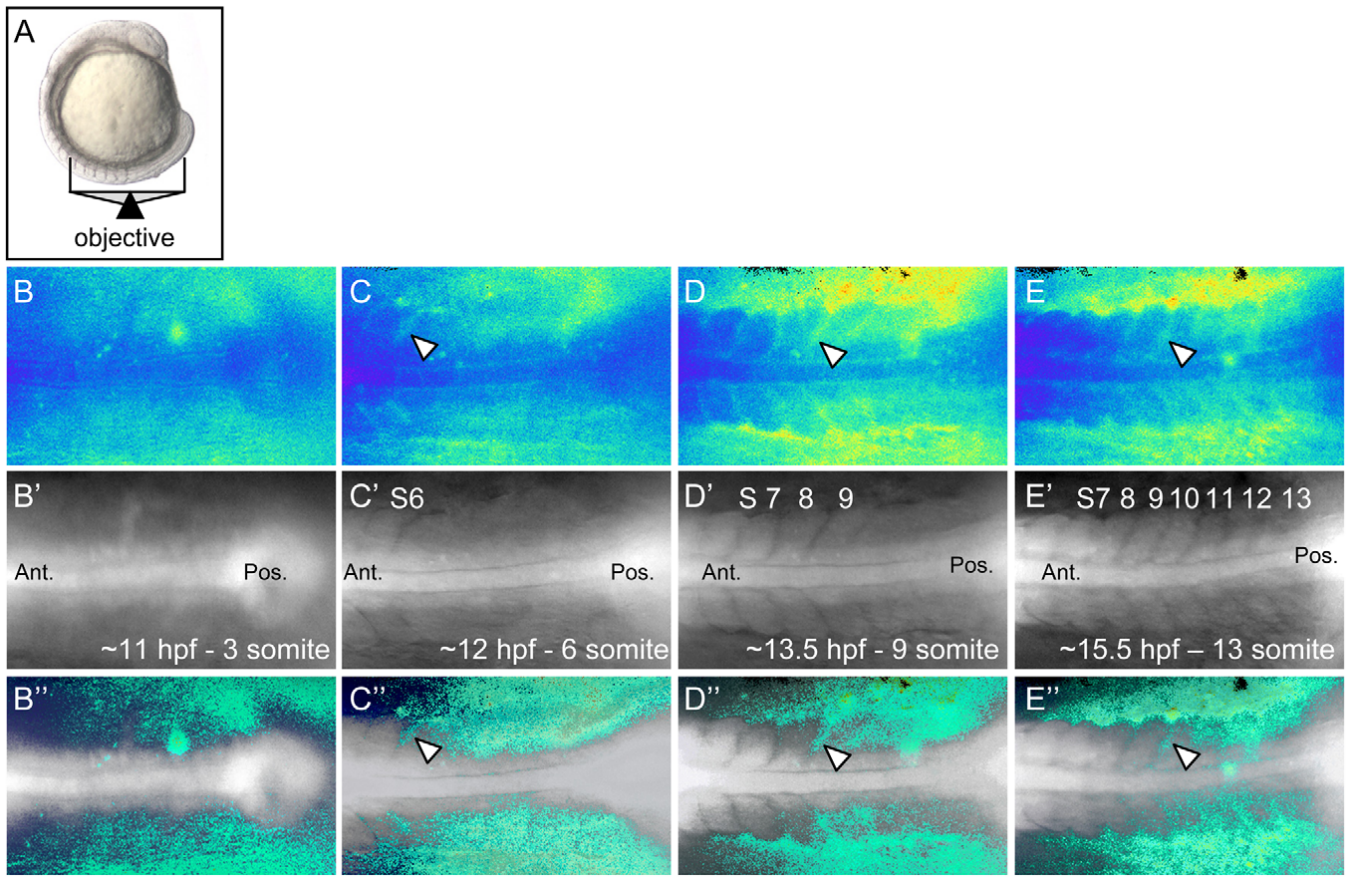
Ca^2+^ dynamics during zebrafish somitogenesis. Illustration of the position of a 10 somite stage (14 hpf) zebrafish embryo relative to the objective during Ca^2+^ imaging (A). Representative ratio images, pseudocolored with low ratio (low Ca^2+^) represented by blue and high ratio (high Ca^2+^) represented by yellow/red, of 3, 6, 9 and 13 somite stage embryos (B–E respectively). The forming somites and notochord can be identified by the grayscale fluorescence images (B′–E′). Overlay of grayscale and ratio images illustrate the regions of Ca^2+^ release activity relative to morphology (B″–E″). Arrowheads indicate areas of sustained Ca^2+^ activity between forming somites. Ant. = Anterior, Pos. = Posterior and S = somite number.

The functional requirement of *rgs3* during anterior-posterior axis extension and the finding that over-expression of *rgs3* is sufficient to inhibit *wnt5*b-induced Ca^2+^ signaling, raised the possibility that *rgs3* may negatively modulate Ca^2+^ release dynamics during somitogenesis. In fact, Ca^2+^ signals along the trunk of zebrafish embryos during somitogenesis have been described using the bioluminescent Ca^2+^ reporter R-aequorin [12], [51], [52]. In order to compare changes in Ca^2+^ release dynamics upon *rgs3* manipulation, we performed a detailed analysis of endogenous Ca^2+^ release in tissues that express both *wnt5b* and *rgs3*. To this end, we utilized Fura-2 imaging to monitor Ca^2+^ activity with a focus on the developing somites and tailbud in either a dorsal (Figure 4A) or a lateral (Figure S2A) orientation. The pseudocolored ratio image provides a spatial and temporal measurement of Ca^2+^ dynamics with low Ca^2+^ represented by blue and high Ca^2+^ represented by yellow/red. Representative pseudocolored ratio images from a time-lapse series of Ca^2+^ measurements (Video S1), spanning the 3–13 somite stages are shown (Figure 4B–4E). The notochord and forming somites can be identified in the grayscale fluorescence images (Figure 4B′–4E′). Overlay of grayscale and ratio images illustrate the regions of increased Ca^2+^ levels relative to morphology (Figure 4B″–4E″).

**Figure 5:**
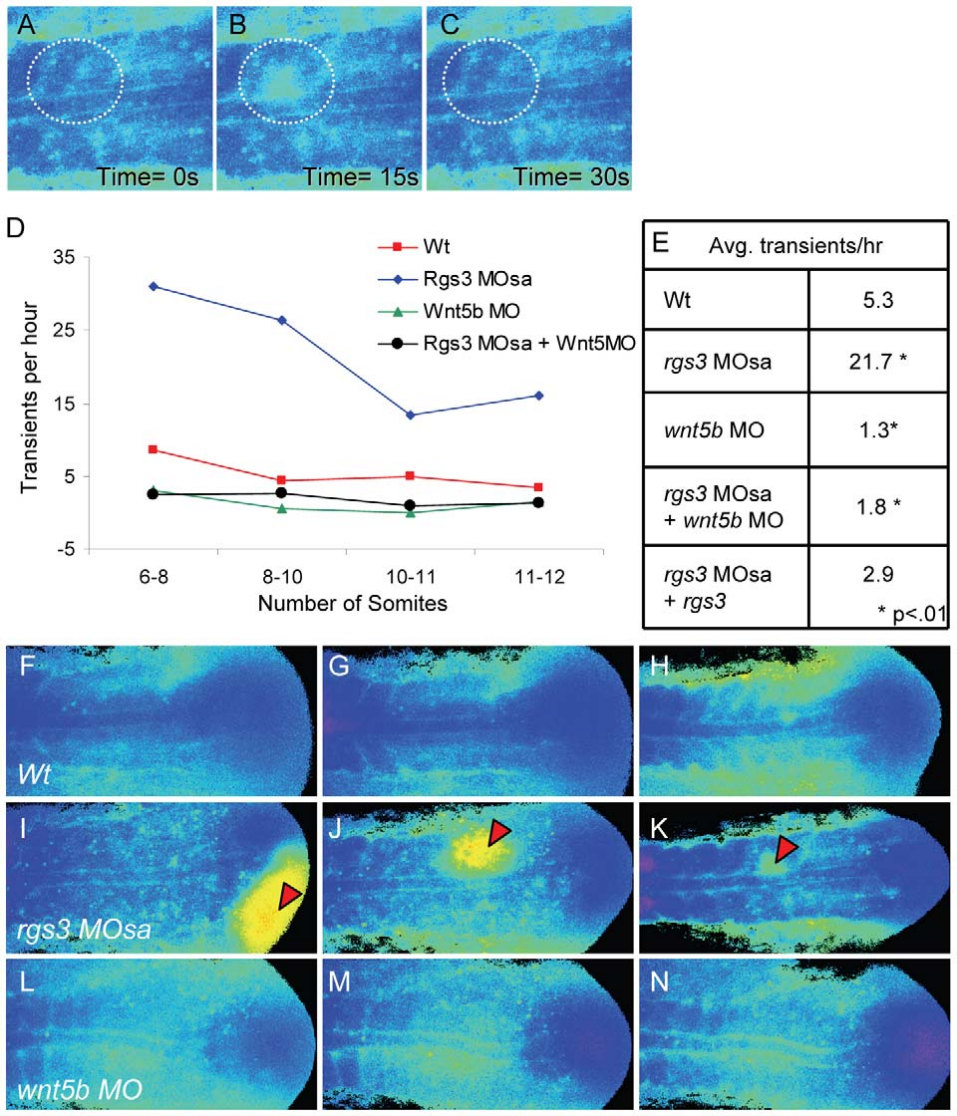
rgs3 impacts segmentation stage Ca^2+^ dynamics. Zebrafish embryos injected with Fura-2 were oriented in a dorsal posterior view. Representative ratio images, pseudocolored with low Ca^2+^ represented by blue, and high Ca^2+^ represented by yellow/red (A–C,F–N). During somitogenesis, Ca^2+^ transients are identified as a local short-lived increase in intracellular Ca^2+^ levels. A region of interest (ROI) is noted by a dashed circle highlighting a representative Ca^2+^ transient (A–C). In the ROI from time 0s to time 15s, an increase in Ca^2+^ levels is observed (B) that subsides by time 30s (C). The number of transients as a function of developmental age (D). Table depicting the average number of Ca^2+^ transients per hour from 6 to 12 somite stage for each treatment (E). Representative ratio images of 5 somite stage (F), 7 somite (G) and 10 somite stage (H) wt embryos taken from Video S1. Representative ratio images of 5 somite (I), 7 somite (J) and 10 somite stage (K) rgs3 MOsa injected embryo taken from Video S2. Representative ratio images of 5 somite (L), 7 somite stage (M) and 10 somite stage (N) wnt5b MO injected embryo taken from Video S4. Red arrowheads indicate large Ca^2+^ transients in *rgs3* morphant embryos (I–K) that are not observed in wt (F–H) or *wnt5b* morphant embryos (L–N).

Ca^2+^ release activity during somitogenesis is dynamic with sustained Ca^2+^ levels in the presomitic mesoderm, lateral to the somite forming region and flanking the midline/notochord (Figure 4B″–4E″). As somitogenesis proceeds, sustained Ca^2+^ levels appear distinctly between the somites (Figure 4C″–4E″, arrowheads). In addition, we observe localized short-lived increases in Ca^2+^ release (referred to as transients). To demonstrate a transient, a region of interest (ROI) is noted by dashed circle (Figure 5A–5C). In the ROI, an increase in Ca^2+^ is observed from time 0s to time 15s and the local increase subsides by time 30s. Since *rgs3* may function to influence the frequency of Ca^2+^ release, we determined the number of transients as a function of developmental age (Figure 5D). In wt embryos, we observe an average of 5.3 Ca^2+^ transients per hour (n = 3) (Figure 5E). A similar frequency is found when analyzing the data from a lateral view (Figure S2B, S2C, S2D, and S2K).

Having defined endogenous Ca^2+^ release dynamics during somitogenesis, we next determined the impact of *rgs*3 knockdown. From the development of somite 6 to somite 12, *rgs*3 morphants have statistically more Ca^2+^ transients, with an average of 21.7 per hour (n = 3), when compared to wt embryos (Figure 5D and 5E). *rgs*3 morphants have sustained Ca^2+^ levels in the lateral regions similar to wt. However the dynamics within the somite region frequently show initiating transients leading to responses in neighboring cells, resulting in larger areas of increased Ca^2+^ release (Figure 5I–5K, Video S2). These large and robust transients are not observed in wt embryos (Figure 5F–5H, Video S1) or in morphant embryos co-injected with *rgs3* RNA (Video S3). The same dramatic increase in both the frequency of release and amplitude is observed in lateral views as well (Figure S2E, S2F, S2G, and S2K). The change in Ca^2+^ release dynamics in *rgs3* morphants is consistent with a delayed shut-off of G protein signaling, i.e. normally mediated by the GAP activity of Rgs3. These data indicate that during the segmentation period Rgs3 functions to limit the extent and duration of endogenous Ca^2+^ release activity.

Previously, we reported reduced Ca^2+^ release in blastula stage Wnt5b (*pipetail*) genetic mutants [24]. When compared to wild-type embryos, *wnt5b* morphant embryos show a statistically reduced number of Ca^2+^ transients, averaging 1.3 per hour (n = 2) during the segmentation period (Figure 5D–5E, 5L, and 5M; Video S4). A similar decrease in frequency is also observed in a lateral view (Figure S2H, S2I, S2J, S2K). The size and duration of Ca^2+^ transients observed in *wnt5b* morphants are comparable to wt embryos (Video S4). In order to determine if the increased frequency of Ca^2+^ transients associated with *rgs3* knockdown is dependent upon *wnt5b* signaling, we simultaneously knocked down *wnt5b* and *rgs3*. Embryos co-injected with *wnt5b* MO and *rgs3* MO and imaged during the segmentation period show a statistically reduced number of Ca^2+^ transients, 1.8 per hour (n = 5) (Figure 5D–5E). The reduced Ca^2+^ release in the double knockdown is not significantly different than *wnt5b* single knockdown, demonstrating that the *rgs3* morphant phenotype is dependent upon Wnt signaling.

### *rgs3* and *wnt5b* interaction

**Figure 6:**
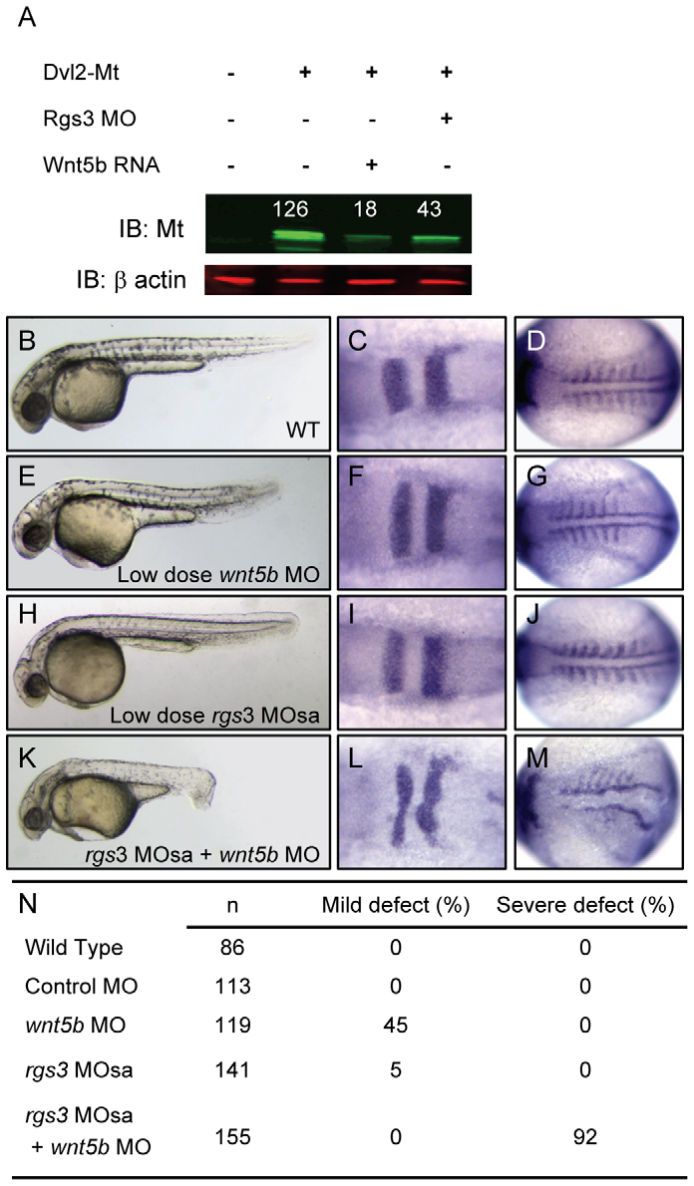
*rgs3* interacts with the Wnt-signaling network. Western analysis demonstrates that Dvl-MT levels are reduced by both *wnt5b* overexpression as well as Rgs3 knockdown (A). Odyssey Infrared Imaging System was used to quantify the relative intensity of Dvl-MT normalized to the β actin loading control and shown as numbers above the IB∶MT bands. Low doses of *rgs3* and *wnt5b* MOs were used to test genetic interaction (B–N). Phenotypes were characterized by morphology (B,E,H,K,N) and the molecular markers *krox20* (C,F,I,L) and *myoD* (D,G,J,M). Lateral images of 34 hpf wt (B), low dose *wnt5b* MO (E), low dose *rgs3* MOsa (H), and *wnt5b* MO+*rgs3* MOsa (K) injected embryos. Dorsal images of 13hpf wt (C,D), low dose *wnt5b* MO (F,G), low dose *rgs3* MOsa (I,J), and *wnt5b* MO+*rgs3* MOsa (L,M) injected embryos. Low dose *wnt5b* MO+*rgs3* MOsa resulted in a 92% penetrance of severe defects which were not observed with low dose *wnt5b* MO or low dose *rgs3* MOsa alone (N).

Studies have shown that increased Wnt/Fz signaling leads to degradation of Dvl [53]–[55]. In addition *Drosophila* genetics places active G protein signaling upstream of Dvl [32]. Therefore, it seemed essential to determine whether Rgs3 plays a role in modulation of Dvl levels. In the absence of an antibody to evaluate Dvl levels, we generated a myc-tagged (MT) form of zebrafish Dvl2 that is readily detected by western blot after injection into embryos (Figure 6A). We find that *wnt5b* co-expression reduced Dvl-MT levels (Figure 6A). Reduction of Rgs3 function, via MO knockdown, also leads to decreased Dvl-MT levels. These data demonstrate that endogenous Rgs3 functions in the non-canonical Wnt pathway upstream of Dvl, thereby functioning to modulate the duration and robustness of Wnt5 signaling. To further explore interaction between Rgs3 and Wnt5b, we defined a low dose for *wnt5b* MO which results in a mild A-P extension phenotype and determined whether *rgs3* enhances or suppresses the *wnt5b* gene knockdown defects. Phenotypes were evaluated by morphology (Figure 6B, 6E, 6H, and 6K) and molecular markers, *krox20* and *myoD* (Figure 6C–6D, 6F, 6G, 6I, 6J, 6L, and 6M). Compared to wt (Figure 6B–6D), low dose *wnt5b* MO (2 ng) results in a mild phenotype (Figure 6E–6G). We next defined a sub-phenotypic dose for *rgs3* MOsa (0.8 ng), which produced a phenotype (Figure 6H–6J) indistinguishable from wt (Figure 6B–6D). Individual injection of low dose *rgs3* MOsa or *wnt5b* MO did not induce any severe defects (Figure 6N). However, *wnt5b* MO (2 ng) combined with *rgs3*MOsa (0.8 ng) resulted in a 92% penetrance of severe defects (Figure 6K–6N). Our Ca^2+^ imaging implicated Rgs3 function in limiting the extent and duration of endogenous Ca^2+^ release activity and that this was dependent upon Wnt5. However, in the presence of low level Wnt5 activity (low-dose MO), partial knockdown of *rgs3* could lead to discordant changes in the frequency and amplitude of Ca^2+^ release result in the dramatic phenotypic penetrance and severity.

## Discussion

These results provide new evidence for an essential role of Rgs3 in modulating the duration of Wnt5b signaling. We show that Rgs3 is necessary for proper gastrulation and somite patterning during zebrafish development. These actions of Rgs3 require its ability to interact with and accelerate the rate of GTP hydrolysis by G proteins, as revealed by studies employing an Rgs3 mutant defective in these activities. We describe endogenous Ca^2+^ release dynamics during somitogenesis. The frequency of Ca^2+^ transients as well as the observation of sustained Ca^2+^activity in the trunk and tail region are consistent with previous reports of Ca^2+^ activity during zebrafish somitogenesis [12], [51], [52], [56]. Of particular significance is the dramatic change in frequency of endogenous Ca^2+^ release upon *rgs3* knockdown.

RGS proteins were identified as negative regulators of G protein signaling in the mid 1990s [57], [58] and the role of G proteins in Wnt/Ca^2+^ signaling was first demonstrated in 1997 [22]. Subsequent reports further implicated G proteins in canonical Wnt signaling [31], [59], [60]. Heterotrimeric G protein activation and inactivation are tightly regulated to provide precise control of the amplitude and duration of G protein signaling. Receptor-stimulated GTP binding activates G proteins, while their intrinsic GTPase activity functions to terminate signaling. RGS proteins by definition accelerate this GTPase activity. Over-expression studies in cell culture [61] and in Xenopus embryos [62] have indicated that specific RGS proteins are sufficient to regulate canonical Wnt signaling. Although G protein signaling is required for normal cell movement during zebrafish gastrulation [11], the role of RGS proteins in noncanonical Wnt signaling has not been investigated. Our current study identifies a member of the RGS protein family that has a direct impact on frequency and amplitude of Wnt5b signaling. We find that Rgs3 activity is sufficient to modulate *wnt5b* induced Ca^2+^ release and this ability requires GAP activity consistent with the known role of G proteins in the activation of Wnt signal transduction pathways [5], [63], [64]. We report the key novel finding that knockdown of Rgs3 results in increased frequency and amplitude of Ca^2+^ release that this dramatic impact on Ca^2+^ dynamics in the somites is dependent upon Wnt5 supporting that Wnt/Ca^2+^ signaling activity is an *in vivo* target of RGS proteins. Moreover, *rgs3* demonstrates a complex genetic interaction with *wnt5b*. *rgs3* is expressed in and near *wnt5b* expressing tissues and we find that combined low doses of *wnt5b* MO and *rgs3* MOsa result in a large penetrance of severe somite defects which is not observed during their individual knockdown. Our data suggest that both the frequency and amplitude of *wnt5b* signaling must be tightly regulated to result in correct cell movement and somite patterning.

Wnt5b modulates both transient Ca^2+^ release activity in small populations of cells, as well as, sustained activity in a large region of cells [16]. While the transient release correlates with limiting β-catenin activity [17], [26], we hypothesize that the sustained activity correlates with polarized cell movement, for example in convergence-extension movements during gastrulation or neural and vascular outgrowth [16]. This concept is supported by vascular outgrowth defects in *pipetail* genetic mutants [65] as well as the observation of a reduction in sustained Ca^2+^ activity at the somite boundaries (data not shown). It is of interest to determine if interactions between *rgs*3 and *wnt5b* influence directed vascular outgrowth.

Modulation of G protein signaling (impacting frequency as well as duration) is likely to influence directed cell migration, vascular development as well as numerous other developmental processes [66]–[68]. Our findings clearly justify the need for further investigations into the role of RGS proteins in this process and other interactions between Rgs3 and Wnt signaling to provide new insights into their mechanistic role in directed cell movement and disease. Our loss of function analysis coupled with rescue and *in vivo* physiological analysis in whole embryos has provided compelling functional insight into the developmental role of RGS proteins in the Wnt signaling network.

## Materials and Methods

### Zebrafish

Adults were maintained in a 14-hour light / 10-hour dark cycle at 28°C. Embryos were collected from natural pairwise matings and staged by hours post fertilization (hpf) at 28.5°C and morphological criteria described in Kimmel et al. [50], [69].

### Zebrafish *rgs*3 point mutants

*rgs*3 (clone IBD5096) was isolated in an expression screen in zebrafish [41] and generously provided by Dr. I. Dawid. MO-resistant *rgs*3 was generated by RT-PCR and directionally cloned (5′-3′) into the pCS2+, pCS2+ myc or pCS2+ Flag expression vector. The Quick Change II site-directed mutagenesis kit (Stratagene) was used to generate an Asparagine (N) to Alanine (A) substitution at amino acid 109 which is located in the RGS domain of Rgs3. Synthetic RNA was then *in vitro* transcribed using SP6 RNA polymerase and the mMessage mMachine system (Ambion).

### Micro-injections

Antisense morpholino oligonucleotides (MO) were designed to target the 5′-UTR/ATG (*rgs3* MO and *rgs3* MOb) to inhibit translation and an intron-exon junction in the RGS domain (*rgs3* MOsa) to alter splicing. As a control *rgs3* MOmm (5 bp mismatch in lowercase letters) was designed (Gene-Tools):

*rgs3* MO 5′-AGTCGGTTCTTCATGTCTTTGGCCC-3′,

*rgs3* MOb 5′-TCTCCGAGAAATCCACCATAGTGTG-3′,

*rgs3* MOsa 5′-CCAGTCCATCTGATGAGGGAGAGAG-3′.

*rgs3* MOmm 5′-TCaCCcAGAAATCCtCCATtGTcTG-3′.

MOs (5–20ng) were pressure-injected into one cell-stage embryos. For low-dose knockdown, 0.8ng *rgs3* MOsa and/or 2 ng *wnt5b* MO [65] were injected into one cell zebrafish embryos. Control *rgs3* MOmm did not produce any phenotype at 25 ng. For rescue, *in vitro*-transcribed MO-resistant *rgs3* (500 pg) was co-injected with 20 ng *rgs3* MO. Injected embryos were characterized by morphological and molecular analysis.

### Whole-mount *in situ* hybridization

Embryos were fixed overnight in 4% paraformaldehyde and dechorionated. Single label WMISH was performed as previously described [24], [70], using digoxigenen (Dig)-labeled or dinitrophenyl (DNP)-labeled antisense and sense RNA probes. Detection was carried out using BM purple (Roche Applied Science). Double label WMISH was performed as previously described [71], using both Dig and DNP-labeled antisense probes. Purple color was developed with AP-conjugated anti-Dig and BM purple (Roche Applied Science), and red color was developed with AP-conjugated anti-DNP and INT RED (Roche Applied Science). Reactions were stopped in phosphate-buffered saline (PBS). Embryos were mounted on bridged coverslips and photographed using a Zeiss Stemi M13 Stereoscope and an Axiocam digital camera.

### Western analysis

To compare levels of MT-Rgs3 and mutant MT-Rgs3, embryos were injected with either *myc-rgs3* or *myc-rgs3^(N109A)^* (750 pg). To investigate Rgs3's impact on Dvl, C-terminal myc tagged zebrafish *dvl2* (300 pg) was injected alone, with *rgs3* MOsa (5ng), with *wnt5b* (250pg), and with both *rgs3* MOsa (5ng) and *wnt5b* (250pg). Injected Embryos were allowed to develop to the appropriate stage (5 hpf and 24 hpf) before incubating in lysis buffer (20 µM Tris, 100µM NaCl, 1µM EDTA, 5% Triton, .5%SDS, 10% Leupeptin and 0.1µM PMSF) at room temperature for 3 minutes. Embryos were then disrupted using a pestle, centrifuged at 13,000 rpm for 10 minutes at 4°C and the clear supernatant containing whole zebrafish protein was collected. Approximately 10µg of protein was loaded in each well and separated with SDS-PAGE gel electrophoresis. Proteins were transferred onto nitrocellulose membrane (Li-Cor) and incubated with the following primary antibodies: mouse anti-myc (1∶2,000; Cell Signaling Technology) and rabbit anti-β actin (1∶2,000; Sigma), and then incubated with the following secondary antibodies: IRDye800 anti-mouse (1∶20,000; Li-Cor) and IRDye680 anti-rabbit (1∶20,000; Li-Cor). The signal was visualized using the Odyssey Infrared Imaging System (Li-Cor).

### Immunohistochemistry

Embryos injected with either *myc-rgs3* or *myc-rgs3^(N109A)^* (200 pg) were fixed 1–3 hrs in 4% PFA/1× PBS at sphere/dome stage. Mouse anti–myc antibody (1∶1,500; Cell Signaling Technology), followed by goat-anti-mouse Alexa488 conjugated secondary antibody (1∶400; Molecular Probes) was used to detect the rgs3 products. Nuclei were identified with 5 µM TO-PRO-3 (Molecular Probes). Embryos were mounted in an animal pole orientation in bridged coverslips and optically sectioned using two-channel imaging on a scanning laser confocal microscope, Leica TCS SP2. Wide-field fluorescence and bright–field images from a Zeiss Stemi M13 Bio Stereoscope were photographed using Axiovision (Zeiss) software and an Axiocam 5000 camera. Images were merged using Adobe Photoshop CS.

### Intracellular calcium (Ca^2+^) imaging

The ratiometric Ca^2+^-sensing dye Fura-2 or Bis-Fura-2 (Molecular Probes) was injected into 1-cell zebrafish embryos. The excitation spectra are different between Ca^2+^ bound Fura-2 (340-nm) and Ca^2+^ free (380-nm) forms. By taking the ratio of the fluorescence intensity at these wavelengths an estimate of intracellular-free Ca^2+^ can be derived. To stimulate Wnt signaling, *in vitro* transcribed *wnt*5b RNA (400 pg) was co-injected with Fura-2 at the one cell stage. *rgs*3 or *rgs*3^N109A^ RNA (400 pg) was unilaterally injected at the 16-cell stage mixed with dextran-conjugated Texas Red (TxR) lineage tracer. TxR distribution was determined by collecting a reference exposure at 540-nm excitation. For cellular blastoderm stage imaging, embryos were oriented in a lateral position in a glass-bottomed dish on a Zeiss axiovert epifluorescence microscope. Image pairs at 340 and 380-nm excitation wavelengths (510-nm emission) were collected at 15-second intervals. Each imaging session collected 300 image pairs. The ratio image, a pixel by pixel match of both excitation wavelengths, is calculated by computer software (RatioTool, Inovision). The sequence of ratio images was processed and the Ca^2+^ fluxes (transients) were determined by a subtractive analog patterned after [72], [73] and described in [13], [43]. The ratio image (340nm, Ca^2+^-saturated and 380nm, Ca^2+^-free) imported for publication is encoded in 8 bits and converted to pseudocolor with low ratio (low Ca^2+^) represented by blue and high ratio (high Ca^2+^) represented by yellow/red.

For somite imaging, 2–6 somite stage embryos were oriented in low melt agarose (0.4%) in a dorsal or lateral orientation. Time courses collected images pairs until 12–15 somite stage at 15-second intervals (Approximately 1000 images pairs). Image pairs were converted to ratio images as described above. Sequential ratio images were manually analyzed for changes in Ca^2+^ transients. Somite stage Ca^2+^ transients were defined as a localized increase in Ca^2+^ which persists no longer than thirty seconds.

### Statistical analysis

Calculations for MO rescue experiments were made using the Fisher's exact test and the two-tailed p-value was reported. Calculations for analysis of somite stage Ca^2+^ transients in morphant embryos were made using One-Way Analysis of Variance (one-way ANOVA) with Tukey HSD test p-values reported.

## Supporting Information

Figure S1 *rgs3* expression is adjacent and overlapping with wnt5b, related to Figure 1. Temporal and spatial expression of *rgs3* compared to *wnt5b* in zebrafish development. Whole Mount *In Situ* Hybridization was utilized to compare the spatial expression of *wnt5b* to *rgs3*. WMISH of 14hpf (A–D) and 24hpf (E–F) Wt embryos. Lateral (A–C and E) and dorsal (D and F) views illustrate that *rgs3* is expressed in the developing somites and posterior tail (A–E). Co localization of *wnt5b* and *rgs3*, determined by double label WMISH with *wnt5b* (red) and *rgs3* (blue), shows adjacent and overlapping expression domains around Kupffer's vesicle (C) and in the tailbud (D). Double label WMISH with *rgs3* (blue) and *engrailed1* (red) highlight that *rgs3* is expressed in the midbrain/hindbrain boundary (E). Sense probes (negative control) gave no specific hybridization signal.
(1.19 MB TIF)

Figure S2 *rgs3* impacts segmentation stage calcium dynamics, related to Figure 5. Zebrafish embryos injected with Fura-2 oriented in a lateral posterior view (A) with a focus on the developing somites and tail (boxed region). Ratio images, pseudocolored to represent low Ca^2+^ as blue and high Ca^2+^ as yellow/red (B–J). Representative ratio images of 6 somite stage (B, E and H), 8 somite stage (C, F, and I) and 10 somite stage (D, G and J) embryos. Arrowheads indicate large Ca*2+* transients in rgs3 morphant embryos (E–G) that are not observed in Wt (B–D) or wnt5b morphant embryos (H–J). The number of Ca^2+^ transients per hour observed in embryos oriented in a lateral posterior view from 6 to 12 somite stage is represented function of developmental age is represented graphically (K).
(2.07 MB TIF)

Table S1 Rescue efficiency of *rgs3* knockdown, related to Figure 2.
(0.04 MB DOC)

Video S1 Wt, Ca^2+^ dynamics during zebrafish somitogenesis, related to Figure 5. Time-lapse movie (Wt zebrafish embryo oriented in a dorsal posterior view) consisting of pseudocolored ratio images derived from image pairs (340 and 380-nm excitation wavelengths) collected in 15-second intervals over a two hour period with low Ca^2+^ represented by blue and high Ca^2+^ represented by yellow/red.
(2.58 MB AVI)

Video S2 rgs3 morphant, Ca^2+^ dynamics during zebrafish somitogenesis, related to Figure 5 Time-lapse movie (*rgs3* morphant zebrafish embryo oriented in a dorsal posterior view) consisting of pseudocolored ratio images derived from image pairs (340 and 380-nm excitation wavelengths) collected in 15-second intervals over a two hour period with low Ca^2+^ represented by blue and high Ca^2+^ represented by yellow/red.
(2.58 MB AVI)

Video S3 rgs3 morphant rescued with *rgs3* RNA, Ca^2+^ dynamics during zebrafish somitogenesis, related to Figure 5. Time-lapse movie (rgs3 morphant rescued with *rgs3* RNA zebrafish embryo oriented in a dorsal posterior view) consisting of pseudocolored ratio images derived from image pairs (340 and 380-nm excitation wavelengths) collected in 15-second intervals over a two hour period with low Ca^2+^ represented by blue and high Ca^2+^ represented by yellow/red.
(1.23 MB AVI)

Video S4 wnt5b morphant, Ca^2+^ dynamics during zebrafish somitogenesis, related to Figure 5. Time-lapse movie (wnt5b morphant zebrafish embryo oriented in a dorsal posterior view) consisting of pseudocolored ratio images derived from image pairs (340 and 380-nm excitation wavelengths) collected in 15-second intervals over a two hour period with low Ca^2+^ represented by blue and high Ca^2+^ represented by yellow/red. Double label WMISH with *rgs3* (blue) and *engrailed1* (red) highlight that *rgs3* is expressed in the midbrain/hindbrain boundary (E).
(2.58 MB AVI)
