# Correction: Regulator of G Protein Signaling 3 Modulates Wnt5b Calcium Dynamics and Somite Patterning

**DOI:** 10.1371/annotation/ab818270-c8c1-4d5d-8acf-ebad6e1d5c26

**Published:** 2010-08-20

**Authors:** Christina M. Freisinger, Rory A. Fisher, Diane C. Slusarski

Some information was omitted from the authors' funding statement. The correct funding statement is: This work was funded by NIH grants to DCS and RAF (http://www.nih.gov) and an AHA predoctoral fellowship to CMF (http://www.americanheart.org). The funders had no role in study design, data collection and analysis, decision to publish, or preparation of the manuscript.

