# Supplementary figures and images for: Regulator of G Protein Signaling 3 Modulates Wnt5b Calcium Dynamics and Somite Patterning
Source: PLoS Genet. 2010 Jul 8;6(7):e1001020. doi: 10.1371/journal.pgen.1001020 (PMC2900303; doi:10.1371/journal.pgen.1001020)

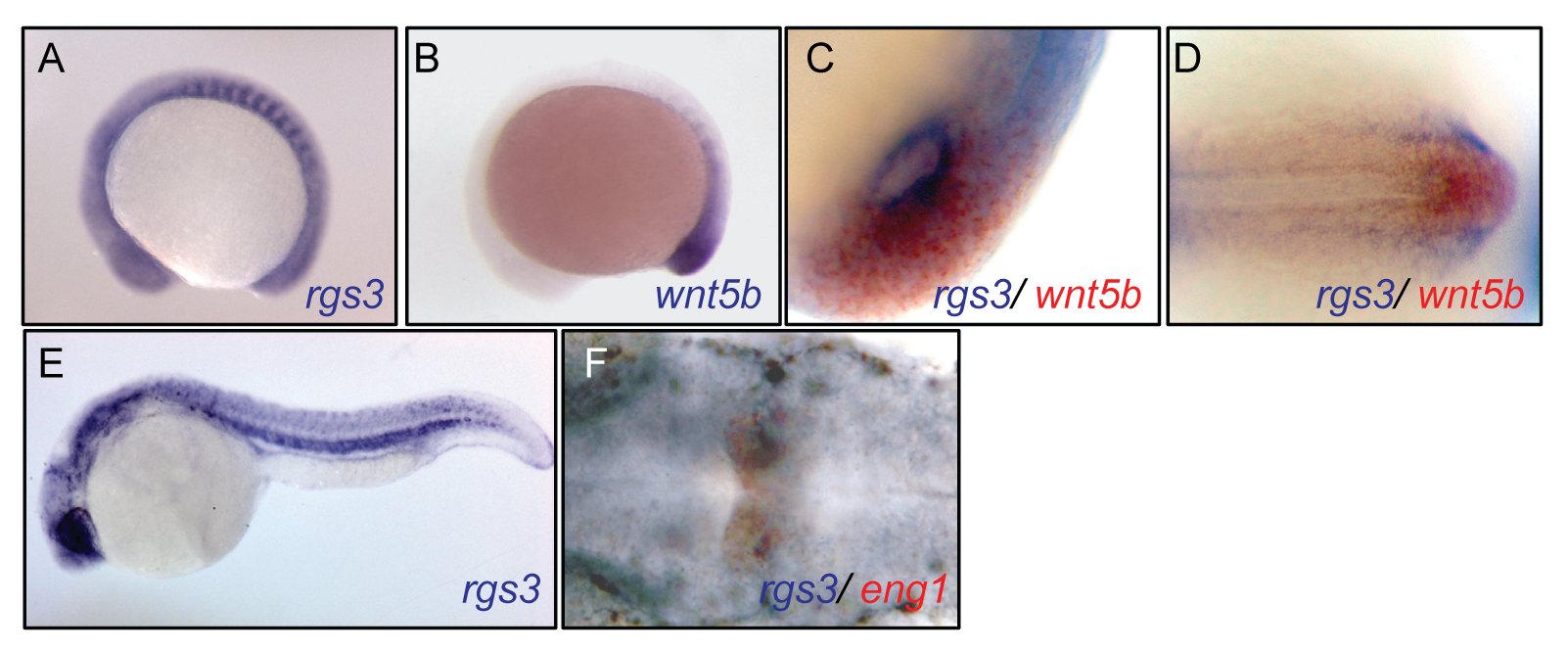

Supplement: Figure S1 — rgs3 expression is adjacent and overlapping with wnt5b, related to Figure 1. Temporal and spatial expression of rgs3 compared to wnt5b in zebrafish development. Whole Mount In Situ Hybridization was utilized to compare the spatial expression of wnt5b to rgs3. WMISH of 14hpf (A–D) and 24hpf (E–F) Wt embryos. Lateral (A–C and E) and dorsal (D and F) views illustrate that rgs3 is expressed in the developing somites and posterior tail (A–E). Co localization of wnt5b and rgs3, determined by double label WMISH with wnt5b (red) and rgs3 (blue), shows adjacent and overlapping expression domains around Kupffer's vesicle (C) and in the tailbud (D). Double label WMISH with rgs3 (blue) and engrailed1 (red) highlight that rgs3 is expressed in the midbrain/hindbrain boundary (E). Sense probes (negative control) gave no specific hybridization signal. (1.19 MB TIF) [file pgen.1001020.s001.tif]

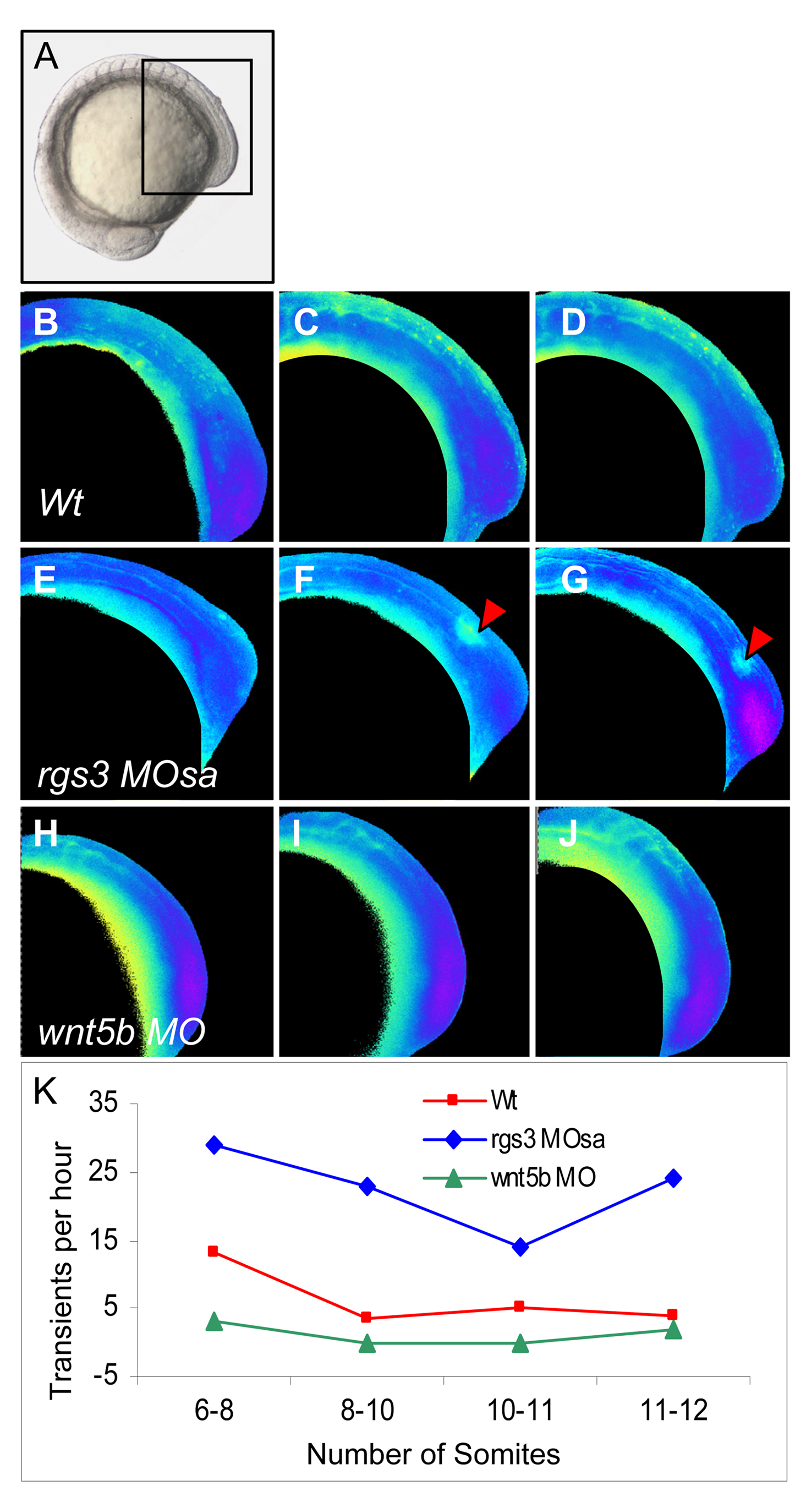

Supplement: Figure S2 — rgs3 impacts segmentation stage calcium dynamics, related to Figure 5. Zebrafish embryos injected with Fura-2 oriented in a lateral posterior view (A) with a focus on the developing somites and tail (boxed region). Ratio images, pseudocolored to represent low Ca2+ as blue and high Ca2+ as yellow/red (B–J). Representative ratio images of 6 somite stage (B, E and H), 8 somite stage (C, F, and I) and 10 somite stage (D, G and J) embryos. Arrowheads indicate large Ca2+ transients in rgs3 morphant embryos (E–G) that are not observed in Wt (B–D) or wnt5b morphant embryos (H–J). The number of Ca2+ transients per hour observed in embryos oriented in a lateral posterior view from 6 to 12 somite stage is represented function of developmental age is represented graphically (K). (2.07 MB TIF) [file pgen.1001020.s002.tif]
