# Supplementary material for: Regulator of G Protein Signaling 3 Modulates Wnt5b Calcium Dynamics and Somite Patterning
Source: PLoS Genet. 2010 Jul 8;6(7):e1001020. doi: 10.1371/journal.pgen.1001020 (PMC2900303; doi:10.1371/journal.pgen.1001020)
Supplement: Table S1 — Rescue efficiency of rgs3 knockdown, related to Figure 2. (0.04 MB DOC) [file pgen.1001020.s003.doc]

**Table S1: Rescue efficiency of *rgs*3 knockdown, related to Figure 2**

Morphological and Molecular Analysis of *rgs3* KD and rescue. Morphological, evaluated at 48 hpf and molecular analysis, of MyoD and Krox20 expression domains at 13-16 hpf

|  | **Morphological Analysis** | | | **MyoD/Krox20 WMISH** | | |
| --- | --- | --- | --- | --- | --- | --- |
| **Wild Type** | **n** | **% Wt-like** | **% Somite defects** | **n** | **% Wt-like** | **% Expanded Somites** |
| **Control MO** | 175 | 100 | 0 | 75 | 100 | 0 |
| ***rgs3* MO** | 140 | 100 | 0 | 43 | 100 | 0 |
| ***rgs3* MO + *rgs3*** | 340 | 38 | 62 | 50 | 41 | 59 |
| ***rgs3* MO + *rgs3*N109A** | 152 | 71 | 29 | 35 | 72 | 28 |
| **Wild Type** | 207 | 42 | 58 | 40 | 42 | 58 |
